# Irritable Bowel Syndrome in a Population of a Developing Country: Prevalence and Association

**DOI:** 10.7759/cureus.8112

**Published:** 2020-05-14

**Authors:** Abdul Latif, Farooque Aziz Memon, Mansoor Asad

**Affiliations:** 1 Gastroenterology, Avicenna Medical College, Lahore, PAK; 2 Gastroenterology, Jinnah Postgraduate Medical Centre, Karachi, PAK; 3 Gastroenterology, Liaquat National Hospital and Medical College, Karachi, PAK

**Keywords:** irritable bowel syndrome, abdominal pain, bowel disorder

## Abstract

Introduction: Irritable bowel syndrome (IBS) is included as one of the functional gastrointestinal (GI) related disorders presenting with pain in abdomen of chronic duration in the absence of any identifiable organic cause. Bloating, distension of abdomen, and defecation in disordered manner are other features commonly associated with IBS. It is a complex disorder with mixed physiological, psychological, and social interactions. The present study was carried out with the aim to determine the frequency of IBS in patients presenting with abdominal pain in a population of a developing country.

Materials and methods: A descriptive cross-sectional study was undertaken at the outpatient department of a large tertiary care hospital. Patients of either gender aged between 20 and 50 years presenting with abdominal pain were included. The diagnosis of IBS was established using Rome III criteria. Prevalence of IBS was calculated and confounding factors such as age, gender, marital status, duration of abdominal pain, and economic status were stratified to see their effect on outcome variable by applying chi-square test and taking p-value of <0.05 as significant.

Results: A total of 186 patients were included in this study. Some 100 (53.76%) were male and 86 (46.24%) were female. Mean age of the patients was 33.67 ± 7.08 years. Some 58.6% were married and 41.4% were single. Some 61.83% had mild abdominal pain and 38.17% had moderate pain. Out of the total 186 patients, the frequency of IBS was found to be 35.48%. There was no significant effect of confounding variables such as age, gender, duration of abdominal pain, marital status, and economic status on IBS.

Conclusion: In our study the frequency of IBS in patients with abdominal pain is high with no significant effect of gender, marital status, economic status, and duration of abdominal pain on IBS.

## Introduction

Irritable bowel syndrome (IBS) is a functional bowel disorder without any organic identifiable etiology, and presents with pain or discomfort in abdomen which is associated with symptom of defecation or as a result of change in bowel habits. Other features that are usually and commonly associated with IBS include a feeling of bloating, distension of abdomen, and defecation in disorderly manner [1]. Its symptoms include abdominal pain and altered bowel habits. It is responsible for missed work days almost similar to that of common cold [2]. Various varieties of IBS exist and almost all of them involve abdominal pain of unexplained etiology, which has a great impact on a patient’s quality of life (QoL) [3]. Recently, a panel of consensus was created which then updated the Rome criteria. The Rome criteria have been designed to achieve a strategy for standardized diagnosis for its utilization in clinical practice and also in the research [4].

The studies based on population have estimated the IBS prevalence to be around 10%-20% [1]. Moreover, a recent meta-analysis on IBS epidemiology studies has reported a prevalence of approximately 11.2% [5]. According to a study, IBS prevalence of 14% has been reported in Pakistan [6]. Patients suffering from IBS usually tend to have worse health-related quality of life (HRQoL) than patients with certain other etiologies such as gastroesophageal reflux disease (GERD), diabetes, and end-stage renal disease (ESRD) [7].

Irritable bowel syndrome is a highly prevalent gastrointestinal (GI) motility disorder and has chronic duration with bothersome nature of symptoms and it often affects the patients’ QoL and activity level negatively. It also has a substantial economic burden on patients as well as healthcare facilities [8-9]. In one study, prevalence of IBS was assessed in college students and it was found to be approximately 28.3% [10].

Irritable bowel syndrome is a disorder of significant morbidity occurring as a result of interaction of psychological, physiological, and social factors. In a developing country such as Pakistan, very limited data exist regarding prevalence of IBS. The aim of the current study was to determine the frequency of IBS by using the Rome III criteria in outpatients. The purpose of the study was early identification of IBS patients followed by their prompt treatment and to prevent misdiagnosis among such patients.

## Materials and methods

The current descriptive cross-sectional study was carried out from 1st June 2017 till 1st January 2018 at the outpatient department of a large tertiary care hospital. Patients of either gender aged between 20 and 50 years presenting with abdominal pain for a duration of three months or more were included. Abdominal pain was assessed on a visual analog scale (VAS). On VAS scale, the score ranging from 1 to 3 was regarded as mild pain, score ranging from 4 to 6 was regarded as moderate pain, and a score of greater than 6 was regarded as severe pain. For IBS diagnosis as per Rome III criteria, the patient should have had pain in abdomen of recurrent nature or any abdominal discomfort for three days per month at least during past three months with onset of symptoms almost six months before to the diagnosis, having association with two or more of the following features: • Pain that got relieved after defecation. • The onset of pain had an association with a change in frequency of stool passing. • The onset of pain had an association with a change in the form of stool. By taking prevalence of IBS as 14%, margin of error as 5%, and confidence interval (CI) as 95%, the calculated sample size came out to be 186 patients by using the WHO’s sample size calculator for Health Sciences. Nonprobability consecutive sampling technique was undertaken. Patients were excluded if they had any known infectious cause of diarrhea and constipation based the history such as abdominal tuberculosis, celiac disease, or HIV. Patients were also excluded if they were taking laxatives, antibiotics, nonsteroidal anti-inflammatory drugs, chemotherapy, medications causing constipations such as antidepressants, antipsychotics, calcium supplements, diuretics, and iron supplements. Patients having red flag symptoms such as weight loss ≥ 5 kg in a month, hemoglobin level of >10 g/dL, bleeding per rectum (two teaspoon in quantity) were also excluded. Data were analyzed by using statistical package for social sciences (SPSS) version 21. Mean and standard deviation were computed for quantitative variables, i.e. age, duration of abdominal pain. Frequency and percentage were calculated for qualitative variables, i.e. gender, marital status, socio-economic status, severity of abdominal pain outcome variable (IBS). Age of the patients, gender, marital status, status of economy, duration of abdominal pain, and severity of abdominal pain were stratified to see their effect on outcome (IBS) by using chi square test and considering p ≤ 0.05 as significant.

## Results

A total of 186 patients were included in the present study. The average age of the patients was 33.67 ± 7.08 years and median duration of abdominal pain was eight. There were 32.8% patients between age 20 and 30 years, 49.46% between age 31 and 40 years, and 17.74% between age 41 and 50 years. Out of 186 cases, 100 (53.76%) were male and 86 (46.24%) female. Some 58.6% were married and 41.4% were single. Most of the patients were from economic class. Regarding severity of abdominal pain of the patients, 61.83% tolerated mild pain and 38.17% tolerated moderate pain. Baseline characteristics are summarized in Table 1.

**Table 1:** Baseline characteristics of the patients (n=186). ^a^mean ± standard deviation (SD); n, number

	n	%
Age, years	33.67 ± 7.08^a^	
Gender		
Males	100	53.76
Females	86	46.24
Marital status		
Married	109	58.6
Single	77	41.4
Severity of abdominal pain		
Mild	115	61.83
Moderate	71	38.17
Monthly income		
<10,000	7	3.76
10,000-20,000	115	61.83
>20,000	64	34.41
Irritable bowel syndrome		
Present	66	35.48
Absent	120	64.52

Prevalence of IBS in our study was 35.48%. There was no significant effect of confounding variables such as age, gender, duration of abdominal pain, marital status, and economic status on IBS (Table 2).

**Table 2:** Comparison of irritable bowel syndrome with clinical characteristics. *Chi-square test was applied

	Irritable bowel syndrome		Total	p value
Yes n=66	No n=120
Gender				
Male	40(40.0%)	60 (60.0%)	100	0.165^*^
Female	26 (30.2%)	60 (69.8%)	86	
Age group				
20-30 years	22 (36.1%)	39 (63.9%)	61	
31-40 years	34 (37.0%)	58 (63.0%)	92	0.785^*^
41-50 years	10 (30.3%)	23 (69.7%)	33	
Marital status				
Married	39 (35.8%)	70 (64.2%)	109	0.920^*^
Single	27 (35.1%)	50 (64.9%)	77	
Socioeconomic status				
<10,000	1 (14.3%)	6 (85.7%)	7	
10,000-20,000	37 (32.2%)	78 (67.8%)	115	0.920^*^
>20,000	28 (43.8%)	36 (56.3%)	64	
Duration of abdominal pain				
≤6 months	30 (40%)	45 (60%)	75	0.290^*^
>6 months	36 (32.4%)	75 (67.6%)	111	
Severity of abdominal pain				
Mild	44 (38.3%)	71 (61.7%)	115	0.314^*^
Moderate	22 (31%)	49 (69%)	71	

## Discussion

Irritable bowel syndrome included as one of the GI disorders manifested as altered habits and chronic abdominal pain without any identifiable organic etiology. Among GI related abnormalities, it is the most frequently diagnosed. Almost 30% of the GI referrals are due to this condition [11]. Pain in abdomen is a hallmark of this condition. It is believed to occur as a result of effect on brain and GI system. It is believed that smooth muscle activity in a disorderly manner together with alteration in sensory pathway may result in enhanced visceral perception [12]. People who are most affected by IBS are the ones usually presenting with the main symptom of pain in abdomen [13]. Moreover, this symptom is a major predictor of severity of IBS as well as utilization of health care [14].

A study conducted on 755 patients suffering from IBS showed that the abdominal pain was a main factor that was a predictor of patient perceived severity. Among other factors affecting the severity, straining with defecation and myalgias were also included [15]. The study also showed that abdominal pain correlated with HRQoL [15]. It is reported that almost all age group patients, including children, are affected by IBS [16].

Our study was conducted on 186 patients and included both males and females. The results of our study have shown a high prevalence of IBS in our population. We reported IBS prevalence of 35.4%. Our reported prevalence is higher than the one reported in Lebanese population [17]. A study from Japanese population also reported a lower prevalence of 18.6% as compared to our population [18]. Another regional study from India has reported a lower prevalence of 4% [19]. A high prevalence in our study could be attributed to genetic and ethnic differences. Moreover, a difference in sample size could also be responsible in this discrepancy.

In our study, males had higher presence of IBS as compared to females, but there was no significant difference noted. According to a previous study, IBS was significantly higher in females [17]. This could be related to the fact that sex hormones may have a role in the development of IBS. Moreover, after infection, IBS tends to be higher in females and according to one study the odds of developing post-infectious IBS are higher in females [20]. Differences in gender are well established in IBS. Younger patients and women are likely to be diagnosed with IBS [5]. Another study showed no significant gender difference in IBS [21]. It has also been postulated that IBS is underdiagnosed in Asian countries and it may increase in prevalence as a result of change in dietary habits and infectious risk factors [22].

Initially, IBS was diagnosed based on Rome II criteria after a careful evaluation of patient's medical history and general physical examination to look for any alarming or red flag features. Recently, the Rome III criteria have been published [23]. The Rome III criteria made some changes over the previous criteria. This functional disorder is currently diagnosed based on a characteristic symptom cluster in the absence of detectable structural abnormalities. IBS is no longer a diagnosis if any organic cause is established/found for the abdominal pain. Abdominal pain in patients with IBS is often described as a cramping sensation, which in some cases may be quite severe [24]. Emotional stress or eating may increase the pain severity. Defecation may help in its relief [23].

Few limitations are present in this study. This was a single institute study. Moreover, in this study the educational status was not considered. According to a study, IBS is usually found more in patients with higher education status [25] and this requires evaluation in further studies. Another limitation of our study was that HRQoL was not evaluated. Patients with IBS tend to have a negative impact on their HRQoL and its determination in developing country population is essential so that measures can be taken to improve it.

## Conclusions

Irritable bowel syndrome has high frequency in our population. Although the frequency of IBS was high in males, there was no statistically significant difference observed. Moreover, no significant difference was observed with age group, marital status, socioeconomic status, and duration of abdominal pain.
